# Histone chaperone Spt16p is required for heterochromatin mediated silencing in budding yeast

**DOI:** 10.1007/s13238-017-0485-4

**Published:** 2017-11-14

**Authors:** Xiaowei Yan, Jiayi Yang, Jiawei Xu, Jianxun Feng, Qing Li

**Affiliations:** 1 State Key Laboratory of Protein and Plant Gene Research, School of Life Sciences and Peking-Tsinghua Center for Life Sciences, Peking University, Beijing, 100871 China; 2 Department of Cellular and Molecular Pharmacology, Howard Hughes Medical Institute, University of California, San Francisco, San Francisco, CA 94158-2517 USA

**Dear Editor**,

Heterochromatin, the highly-condensed nucleosome arrays in chromosomes, plays an important role in regulating multiple cellular events including transcriptional silencing and chromosome segregation (Allis and Jenuwein, 2016). Aberrant heterochromatin formation is linked to tumor progression, as well as other severe physiological disorders (Hahn et al., 2010). However, the mechanism by which factors contribute to heterochromatin formation remains elusive.

In *Saccharomyces cerevisiae*, heterochromatin forms at two hidden mating-type loci (*HM* loci, namely *HMR* and *HML*), telomeres and rRNA-encoding DNA (rDNA) region (Grunstein and Gasser, 2013). Besides the four silent information regulators, Sir1p–Sir4p, it was reported that histone chaperones are also required for heterochromatin formation. For instance, H3–H4 chaperones chromatin assembly factor 1 (CAF-1), anti-silencing function protein 1 (Asf1p), histone transcription regulator 1 (Hir1p), and regulator of Ty1 transposition protein 106 (Rtt106p) are important for heterochromatin silencing (Enomoto and Berman, 1998; Huang et al., 2005; Huang et al., 2007; Sharp et al., 2001). Genetic studies revealed two parallel pathways among these histone chaperones: Asf1p, Hir1p, and Rtt106p seem to mediate a genetically distinguishable silencing pathway from CAF-1. Interestingly, cells deficient in both pathways are only partially defective in heterochromatin silencing, which suggests other factors might also be involved (Huang et al., 2007).

Histone chaperone complex FACT (facilitates chromatin transcription/transaction), consisting of two essential subunits, Spt16p and Pob3p (SSRP1 in humans), plays a critical role in transcription, replication, and other chromatin based processes in eukaryotes (Formosa, 2012). While both Spt16p and Pob3p are essential in *S*. *cerevisiae*, Pob3p is dispensable in *Schizosaccharomyces pombe*, and contributes to heterochromatin silencing (Lejeune et al., 2007). However, whether FACT also has a role in heterochromatin silencing in *S*. *cerevisiae* remains to be determined. Here, we report that Spt16p functions redundantly with CAF-1 and Rtt106p in heterochromatin silencing and is required for heterochromatin formation at both mating-type loci and telomeres in budding yeast.

To identify other histone chaperones that might function in heterochromatin silencing, we performed a small-scale candidate gene screen using a GFP-based reporter gene silencing assay (Fig. 1A). The expression of GFP gene inserted at the silent *HMR* locus (*hmr*::*GFP*) was used to monitor the degree of silencing by quantifying the percentage of yeast cells expressing GFP (Fig. 1A). Yeast cells harboring double deletion of *CAC1* (the large subunit of histone chaperone CAF-1) and *RTT106* was made as a query strain (Fig. 1B). Consistent with prior studies, 47.5% of *cac1*Δ*rtt106*Δ cells lost GFP silencing while only 0.4% wild-type cells expressed GFP, likely from the background. As a positive control, *sir3*Δ almost completely abolishes cells’ ability in heterochromatin silencing (99.8%). Interestingly, *spt16-m*, a newly identified Spt16p mutant allele in our group, dramatically exacerbates the phenotype of *cac1*Δ*rtt106*Δ (77.3%) (Fig. 1C). The *spt16-m* allele contains two amino acids substitution (*K692AR693A*) in its tandem pleckstrin homology (PH) domain and displays a subtle defect in transcription initiation but a substantial defect in DNA replication-coupled nucleosome assembly (Yang et al., 2016). Thus, this result indicates that Spt16p may also be involved in heterochromatin silencing.

**Figure 1:**
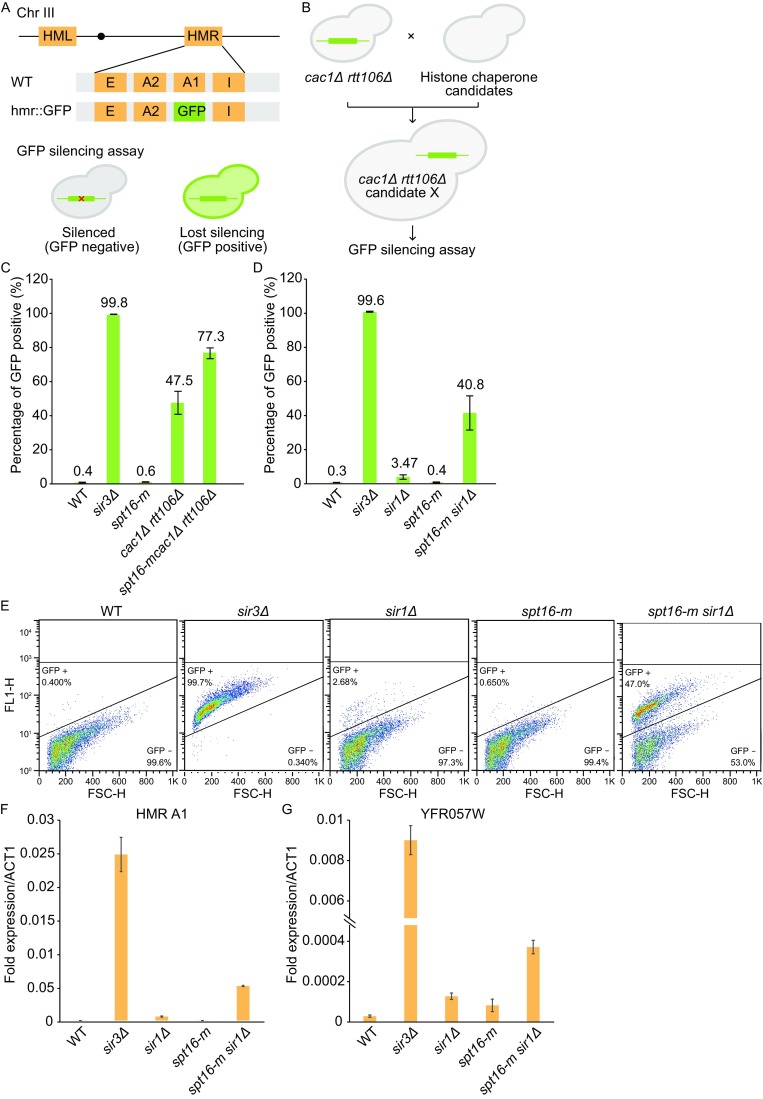
**Histone chaperone Spt16p is required for heterochromatin silencing in** ***S***. ***cerevisiae***. (A) A scheme for the GFP silencing assay. Top: A diagram of yeast chromosome III (Chr III) in either wild-type (WT) strain or strain used for GFP silencing assay (*hmr*::*GFP*). In WT cells, *HMR* contains a silenced copy of mating-type gene including *HMR A1* (*A1*) and *HMR A2* (*A2*), which are surrounded by two silencers, silencer E (E) and silencer I (I). In *hmr*::*GFP* cells, *A1* gene is replaced with a gene encoding GFP as a reporter for silencing assay. Bottom: Cells lost transcriptional silencing will express GFP and become GFP positive during flow cytometer analysis (FACS). (B) Screening strategy for searching histone chaperone that functions in parallel with CAF-1 and Rtt106p in heterochromatin silencing at *HMR* locus. A series of yeast strains containing defects in different histone chaperones were crossed with *cac1*Δ*rtt106*Δ *hmr*::*GFP* query strain to make triple mutant strains, which were then analyzed with GFP silencing assay. (C) Silencing at *HMR* locus is reduced more dramatically in cells deficient in *spt16-m cac1*Δ*rtt106*Δ triple mutant cells than in *cac1*Δ*rtt106*Δ double mutant cells. Expression of the GFP gene at the *HMR* locus in each strain was measured by FACS. The percentage of GFP expressing cells was calculated based on the average of three independent experiments. Error bars: standard deviations (SDs) calculated from three biological repeats. (D) Silencing at *HMR* locus is reduced more dramatically in *spt16-m sir1*Δ double mutant cells than either of the *spt16-m* or *sir1*Δ single mutant cells. Error bars: SDs calculated from three biological repeats. (E) One representative FACS analysis in Fig. 1D. (F) Quantification of *A1* gene expression at the silenced *HMR* locus. The relative expression levels were analyzed by RT-qPCR and normalized to *ACT1* gene. Error bars: SDs calculated from three technical repeats. (G) Quantification of *YFR057W* expression at the right arm of chromosome VI (VI-R) near telomeric region. The relative expression levels were analyzed by RT-qPCR and normalized to *ACT1* gene. Error bars: SDs calculated from three technical repeats

In yeast, Sir1p is involved in the establishment of heterochromatin silencing at the *HM* loci (Rusche et al., 2003). To dissect the role of Spt16p in heterochromatin silencing in yeast, we first examined the role of Spt16p at the *HMR* locus in combination with *sir1*Δ (Fig. 1D and 1E). Consistent with the published results, *sir1*Δ causes a mild silencing defect (3.47%). GFP silencing appears to be normal in *spt16-m* cells (0.4%). However, *spt16-m* strongly exacerbates the silencing defect of *sir1*Δ (40.8%) (Fig. 1D). Similar synergistic behavior between *SPT16* and *SIR1* was also observed in the background of *cac1*Δ *rtt106*Δ (Fig. S1). These results suggest that Spt16p cooperates with Sir1p, CAF-1, and Rtt106 in heterochromatin silencing at the *HMR* locus.

To quantify the transcriptional activity at the *HMR* locus, we used reverse transcription quantitative PCR (RT-qPCR) to analyze the expression level of *A1* gene that resides within *HMR* region (Fig. 1F). Consistent with the previous observations, *A1* gene is silenced in wild-type cells, but is highly transcribed in *sir3*Δ strain. The expression level of *A1* in *spt16-m sir1*Δ double mutant cells is dramatically higher than that in either *spt16-m or sir1*Δ single mutant cells. Moreover, we also analyzed the expression level of a gene *YFR057W* near the telomere of chromosome VI (Fig. 1G). Although no aberrant expression was detected in *spt16-m* strain, the transcription level has been apparently elevated in *spt16-m sir1*Δ strain, indicating a potential role of Spt16p and Sir1p in telomere silencing. While Sir1p is dispensable for telomeric silencing at the modified telomere (Aparicio et al., 1991), it has also been suggested that *SIR1* contributes to silencing at natural telomeres which contains intact subtelomeric repeat sequences (Pryde and Louis, 1999). Together, our data suggested that the cooperation between Spt16p and Sir1p might be required for efficient silencing at both *HM* loci and telomeres.

In yeast, Sir2p is a nicotinamide adenine dinucleotide (NAD) dependent histone deacetylase, which deacetylates nearby histones to facilitate binding of Sir3p and Sir4p. Subsequently, more Sir2p is recruited through direct interaction with Sir3p and Sir4p to spread Sir proteins (Grunstein and Gasser, 2013). A reduction of Sir2p and Sir3p proteins over heterochromatin region was reported in *cac1*Δ *rtt106*Δ strain (Huang et al., 2007). To examine whether Spt16 affects the binding of Sir2–4p to different silent regions, we performed chromatin immunoprecipitation (ChIP) assay using antibodies against Sir2–4p (Fig. 2A–D). As reported, we found that all the Sir proteins are highly enriched at heterochromatin region rather than euchromatin region in wild-type cells, while *sir3*Δ completely abolishes the Sir proteins binding over the heterochromatin region. Moreover, while *spt16-m* mutation led to a mildly reduction of Sir2–4p binding at heterochromatin region, this mutation in combination with *cac1*Δ *rtt106*Δ led to a dramatic reduction of Sir2–4p binding comparing with either single or double mutant cells (Fig. 2A). This data suggests that Spt16p might function in parallel with CAF-1 and Rtt106p in regulating Sir proteins’ occupancy over heterochromatin regions. Furthermore, we found that the levels of Sir proteins drop dramatically in *spt16-m sir1*Δ strain at both *HMR* locus and telomere region (Fig. 2B and 2C). This dramatic defect is not due to alteration in Spt16p expression or chromatin occupancy in the mutant cells (Fig. S2). To further characterize the defect of heterochromatin formation in *spt16-m* mutant cells, we also analyzed the distribution of all three Sir proteins across *HMR* locus (Fig. 2D) and the histone modifications at *HMR* locus (Fig. 2E). Silencers, including *E* and *I* elements, are genomic regions where heterochromatin formation initiates (Grunstein and Gasser, 2013). In agreement with the silencer function of *HMR-E* and *HMR-I* that recruits Sir proteins and promotes formation of heterochromatin inwards toward the mating-type genes, an “M” shape distribution of Sir proteins was detected in wild-type cells. Compared with wild-type cells, the amount of Sir proteins is comparable in *sir1*Δ or *spt16-m* cells and the similar “M” pattern was observed, indicating that the silencer function is largely maintained in either single mutant strains. By contrast, the M pattern is lost in *spt16-m sir1*Δ strain (Fig. 2D). Acetylation of H4 lysine 16 (H4K16Ac) is low at heterochromatin region to facilitate the Sir proteins binding but is high at euchromatin regions adjacent to heterochromatin to restrict heterochromatin spreading into euchromatin (Grunstein and Gasser, 2013). We found that the level of H4K16Ac at the heterochromatin region is more dramatically enhanced in *spt16-m sir1*Δ double mutant cells compared to either single mutant alone (Fig. 2E, left panel). No obvious alterations was observed in H3K56Ac, an acetylation mark of newly synthesized H3 (Li et al., 2008), which was analyzed by ChIP assays side by side (Fig. 2E, right panel). Together, these data further supports the idea that Spt16p may cooperate with Sir1p and function in regulating the binding of Sir2–4p binding to heterochromatic region.

**Figure 2:**
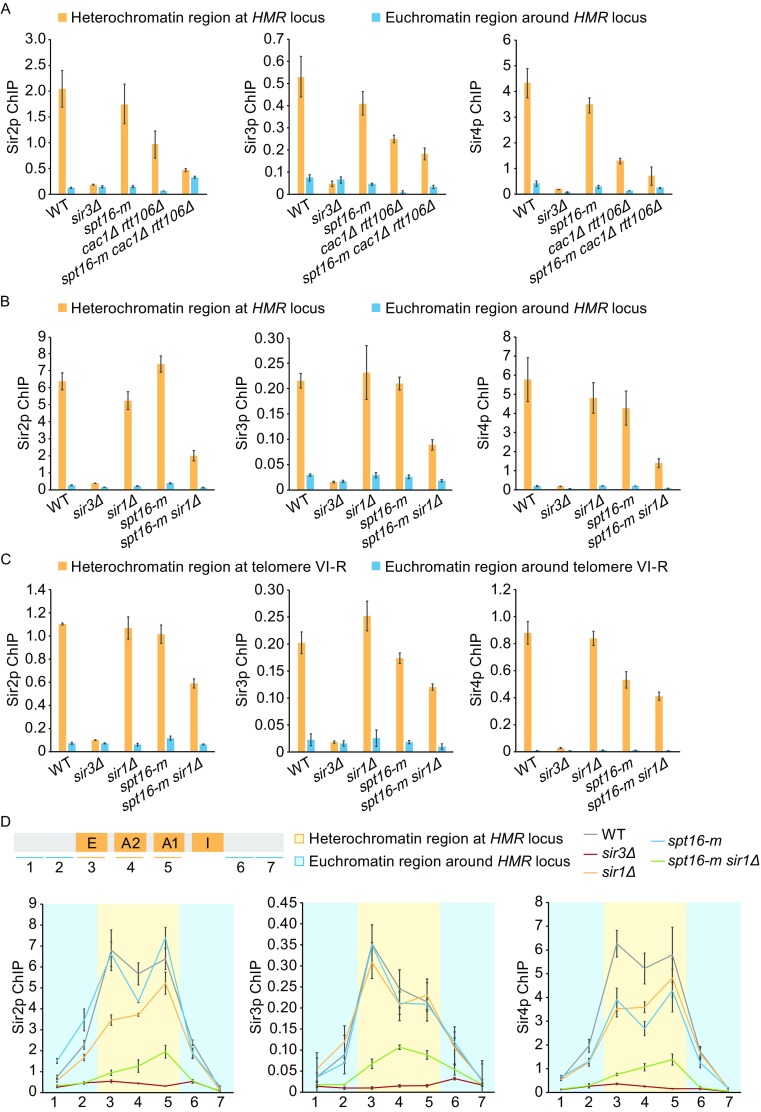

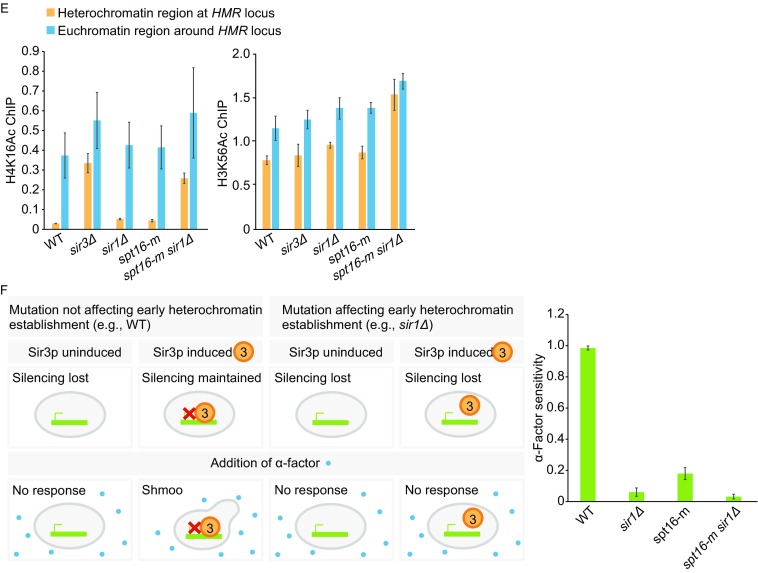
**Spt16p is required for Sir2-4p binding to heterochromatin region and Spt16p cooperates with Sir1p during the establishment of heterochromatin silencing**. (A) Sir2-4p binding at *HMR* locus is dramatically reduced in *spt16-m cac1*Δ *rtt106*Δ triple mutant cells. ChIP assays were performed using yeast strains of the indicated genotype and antibodies against Sir2p, Sir3p, and Sir4p, respectively. The error bars here represent SDs calculated from three technical repeats. The data are presented as the ratio of Sir proteins ChIP signal over input signal. *sir3*Δ strain was used as a control. (B and C) The association of Sir proteins to both *HMR* locus (B) and Chr VI-R telomeric region (C) is dramatically reduced in *spt16-m sir1*Δ double mutant cells. Sir2-4p ChIP was performed as described in (A). (D) The distribution pattern of Sir proteins at *HMR* locus has been changed in *spt16-m sir1*Δ double mutant cells. Top: A schematic diagram of *HMR* locus indicating the location of the primers used in this study. Bottom: The same ChIP assay was performed as described in (A) to map the distribution of Sir proteins across *HMR* locus in the indicated strains. (E) The level of H4K16Ac is increased at the heterochromatin region in *spt16-m sir1*Δ double mutant cells. ChIP assays were performed with antibodies against H4K16Ac and H3K56Ac. The data are presented as the ratio of H4K16Ac or H3K56Ac ChIP signal over input signal, with error bars indicating the SD for three technical repeats. (F) Spt16p has a role in the establishment of heterochromatin silencing. Left: A schematic diagram of the inducible heterochromatin establishment assay. There are two different mating types in *S*. *cerevisiae*: *MATa* and *MATα*. Selected strains for this assay are all *MATa* type cells, which normally only express a-specific genes and inhibit the expression of α-specific genes because of silencing at both *HM* loci. *MATa* cells will respond to the opposite mating pheromone, α-factor, by growing a protrusion called shmoo due to its distinct shape. In the absence of Sir3p (Sir3p uninduced), heterochromatin silencing is lost and such cells fail to respond to α-factor due to the expression of both a-specific and α-specific genes. This defect could be rescued by the induction of Sir3p if there are no other mutations except *sir3*Δ. In cells lacking Sir1p, a protein involved in heterochromatin establishment, establishment of silencing is affected. Green bar: *HM* loci; green arrow: the expression of α-specific genes; red cross: the inhibition of α-specific genes; orange circle with number 3: the presence of Sir3p; blue dot: α-factor. Right: The percentage of shmoo cells after Sir3p induction was counted (*n* = 3 with at least 200 cells for each genotype). Error bars: SDs calculated from three individual experiments

As Sir1p is known to be required for establishment of heterochromatin (Pillus and Rine, 1989), we speculate that Spt16p may also have a role in this stage. To test this, we used a Sir3p-induction system in *sir3*Δ cells to monitor the establishment of heterochromatin at silenced mating-type region (Fig. 2F). Before induction, no Sir3p proteins are present and heterochromatin silencing is completely abolished. Therefore, *MATa* cells will not respond to the mating pheromone α-factor due to lost silencing at yeast silent mating-type locus. Upon induction of Sir3p expression, heterochromatin is re-established in these cells and as a result, the cells will respond to α-factor and form shmoos. Deletion of *SIR1* leads to reduced number of shmoos after Sir3p induction. Notably, comparing with wild-type cells, *spt16-m* also exhibits an apparent defect in shmoo formation after induction of Sir3p. Moreover, *spt16-m* exacerbates the defect of shmoo formation of *sir1*Δ (Fig. 2F). Thus, we conclude that Spt16p has a role in the establishment of heterochromatin silencing and it may function cooperatively with Sir1p during this process.

In summary, we show that histone chaperone FACT is required for the heterochromatin silencing in *S*. *cerevisiae* via the study of its subunit Spt16p. We find that the *spt16-m* mutant aggravates the *HMR* silencing defect in cells lacking both *CAC1* and *RTT106* genes. Moreover, Spt16p functions in parallel with CAF-1 and Rtt106p in regulating Sir proteins’ occupancy. Therefore, besides functioning with CAF-1 and Rtt106p during nucleosome assembly (Yang et al., 2016), we speculate that Spt16p also cooperates with CAF-1 and Rtt106 in heterochromatin formation. Additionally, we find that Spt16p likely functions cooperatively with Sir1p in the establishment of heterochromatin silencing at both telomeres and cryptic mating-type loci. Pob3p has been previously reported to be important for heterochromatin silencing in *S*. *pombe* (Lejeune et al., 2007). Our data suggests that Spt16p might also play a crucial role during heterochromatin silencing in *S*. *cerevisiae*, a highly divergent eukaryotic species from *S*. *pombe*. Therefore, it is likely that FACT’s role in heterochromatin silencing is conserved among eukaryotes.

## Electronic supplementary material

Below is the link to the electronic supplementary material.

Supplementary material 1 (PDF 524 kb)
