# Supplementary material for: Histone chaperone Spt16p is required for heterochromatin mediated silencing in budding yeast
Source: Protein Cell. 2017 Nov 14;9(7):652–8. doi: 10.1007/s13238-017-0485-4 (PMC6019661; doi:10.1007/s13238-017-0485-4)
Supplement: Supplementary file 1 — Supplementary material 1 (PDF 524 kb) [file 13238_2017_485_MOESM1_ESM.pdf]

Materials and Methods

Yeast strains used in this study

Strain	Genotype
LQY375	*MATα sir3Δ*
LQY426	*MATα sir1Δ*
LQY605	*MATa spt16-m*
LQY606	*MATα spt16-m*
LQY747	*MATα spt16-m sir1Δ*
LQY885	*MATα HMR::GFP*
LQY886	*MATa sir3Δ HMR::GFP*
LQY887	*MATa spt16-m HMR::GFP*
LQY888	*MATa spt16-m sir1Δ HMR::GFP*
LQY892	*MATa sir1Δ HMR::GFP*
LQY755	*MATa cac1Δ rtt106Δ HMR::GFP*
LQY1066	*MATa spt16-m cac1Δ rtt106Δ HMR::GFP*
LQY734	*MATa cac1Δ rtt106Δ*
LQY728	*MATa spt16-m cac1Δ rtt106Δ*
LQY3610	*MATa sir3Δ sir3-HA*
LQY3611	*MATa sir3Δ sir1Δ sir3-HA*
LQY3612	*MATa sir3Δ spt16-m sir3-HA*
LQY3613	*MATa sir3Δ spt16-m sir1Δ sir3-HA*

All yeast strains used were of the W303-1 (*leu2-3,112 trp1-1 can1-100 ura3-1 ade2-1 his3-11,15*) genetic background. Standard yeast media (YPD) and manipulations were used throughout this study.

Primers used in this study

Name	Sequence
HMR1-F	TCCTCAGAGATTGTTCTAAAGACC
HMR1-R	TTTTGGTCAACATAAAGTGGCGAG
HMR2-F	AATCTCTAACCCACTAGTACTTA
HMR2-R	AGGATTATTTGACCTTTCTAT
HMR3-F	TCATGTACTAAACTAAAATCAGGG
HMR3-R	CGAGAATCTTCGTAATGCTAA
HMR4-F	GGCGAGGACATTATCTATCAGTAC
HMR4-R	CAGAAAAATAAATCGGCGGATGGG
HMR5-F (hetero)	AAGGATAGCCTTTGAATCAATTTAC
HMR5-R (hetero)	AACTAAAAGAAAAACCCGACTATGC
HMR6-F	ACCAATTCCGCATCTGCAGATTAC
HMR6-R	AGCCGTAAGAGATCTCCGAATAAC
HMR7-F (eu)	CGAGTTCTTCTATATCCGGTGTAC
HMR7-R (eu)	ACCAGGATCTTTTATCTGATAAGC
VI-R-hetero-F	GGACAGATCCTTTCGCATTCCTAC
VI-R-hetero-R	GCGCCTAGTGCAACTAGTGCATAT
VI-R-eu-F	GGATTGACGGGTAACCCTAAAAGG
VI-R-eu-R	GTTGTCATGGCCAATGACCACGAT

Figure 2A, 2B and Figure 2E was performed with *HMR5*-F/R (heterochromatin region) and *HMR7*-F/R (euchromatin region).

GFP silencing assay

GFP silencing assay was performed as previously described (Huang et al., 2007). Briefly, yeast cells harboring *hmr::GFP* in wild type or mutant backgrounds were grown at 26°C and harvested at log phase (OD_{600}=0.4~0.6). After being washed three times with phosphate buffered saline (PBS), cells were resuspended in proper amounts of PBS and analyzed with flow cytometry (BD Calibur). The data collected were later processed using FlowJo 7.6.1.

RT-qPCR assay

Yeast cells were grown and 5 ml of log phase cells were collected for RNA isolation. Standard protocol was followed to isolate RNA from yeast cells. To quantify the expression of specific gene, cDNA was then synthesized from 2 μg of total RNA using Superscript III reverse transcriptase (Invitrogen) according to the manufacturer's protocol, and subsequently the cDNA was analyzed by quantitative PCR (Bio-Rad CFX96).

ChIP assay

The ChIP assay was performed as previously described with minor modifications (Yang et al., 2016). Briefly, log phase yeast cells were grown and cross-linked with paraformaldehyde. After quenching with glycine, yeast cells were collected and lysed using mechanical disruption with glass beads. DNA was then sheared to an average size of 500bp by sonication. After clarification, the supernatant was sedimented with appropriate antibodies overnight. After purification of the chromatin-protein complex using protein-G beads, the DNA was purified and then identified by quantitative PCR (Bio-Rad CFX96).

Inducible heterochromatin establishment assay

A plasmid containing the expression of Sir3p-HA under an inducible *GAL10* promoter was integrated at the *URA3* locus in *sir3*Δ yeast strains as described (Cheng and Gartenberg, 2000; Katan-Khaykovich and Struhl, 2005). Yeast cells were collected after overnight induction of Sir3p-HA in medium containing 2% galactose and then treated with α-factor for 2h at 25℃ to test their α-factor sensitivity. 40× optical objective was used to visualize and count the percentage of shmoo cells.

References

Cheng, T.H., and Gartenberg, M.R. (2000). Yeast heterochromatin is a dynamic structure that requires silencers continuously. Genes Dev 14, 452-463.

Huang, S., Zhou, H., Tarara, J., and Zhang, Z. (2007). A novel role for histone chaperones CAF-1 and Rtt106p in heterochromatin silencing. EMBO J 26, 2274-2283.
Katan-Khaykovich, Y., and Struhl, K. (2005). Heterochromatin formation involves changes in histone modifications over multiple cell generations. EMBO J 24, 2138-2149.
Yang, J., Zhang, X., Feng, J., Leng, H., Li, S., Xiao, J., Liu, S., Xu, Z., Xu, J., Li, D., *et al.* (2016). The histone chaperone FACT contributes to DNA replication-coupled nucleosome assembly. Cell Rep 14, 1128-1141.

Supplementary figures

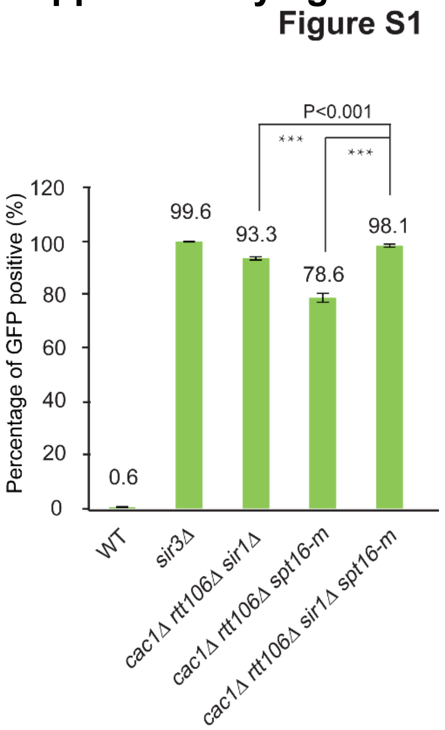

Figure S1. Spt16p functions in parallel with Cac1p and Rtt106p in heterochromatin silencing in *S. cerevisiae*. Silencing defect in *spt16-m cac1Δ rtt106Δ sir1Δ* cells is increased more dramatically than either *spt16-m cac1Δ rtt106Δ* or *cac1Δ rtt106Δ sir1Δ* triple mutant cells. Expression of the GFP gene at the *HMR* locus in each strain of the indicated genotype was measured by FACS. The percentage of GFP expressing cells was calculated based on the average of three independent experiments (n=3). Error bars: standard deviations calculated from three individual experiments. Statistical significance was evaluated based on Student's t-tests (***:P value<0.001).

Figure S2

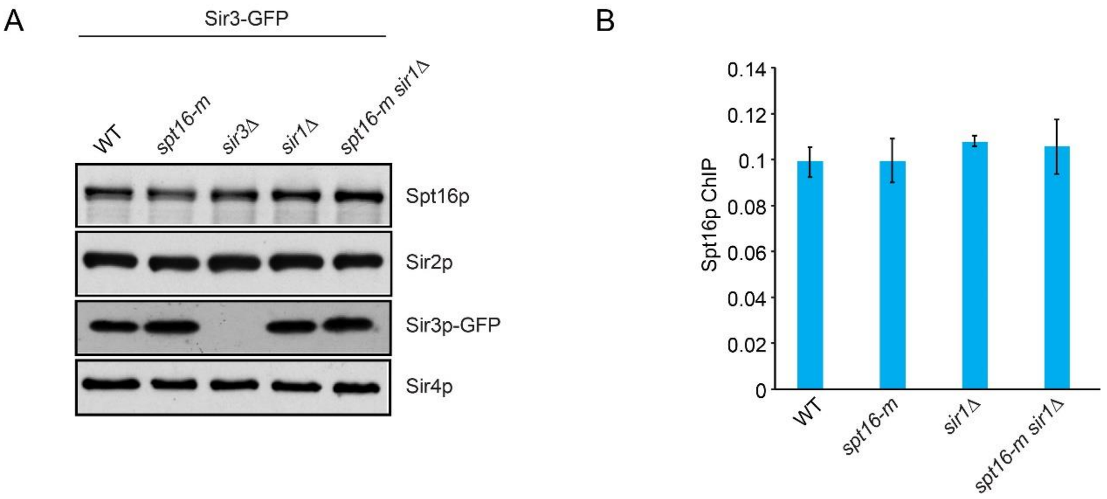

Figure S2. The expression of Spt16p is maintained in *spt16-m sir1Δ* double mutant cells. (A) The expression levels of Spt16p, Sir2p, Sir3p and Sir4p keep unaltered in *spt16-m sir1*Δ double mutant cells. Sir3p was tagged with GFP for detection. Whole cell extracts were prepared and western blotting was performed using antibodies against Spt16p, Sir2p, Sir4p and GFP (for Sir3-GFP). **(B)** The chromatin binding of Spt16p at *HMR* heterochromatin region remains unaffected in *spt16-m sir1*Δ double mutant cells. ChIP assays were performed as described in Figure 2 with primers targeting *HMR5* region, which is labeled in Figure 2D. Error bars represent standard deviations calculated from three technical repeats.
